# Molecular characterization of lung adenocarcinoma from Korean patients using next generation sequencing

**DOI:** 10.1371/journal.pone.0224379

**Published:** 2019-11-25

**Authors:** You Jin Chun, Jae Woo Choi, Min Hee Hong, Dongmin Jung, Hyeonju Son, Eun Kyung Cho, Young Joo Min, Sang-We Kim, Keunchil Park, Sung Sook Lee, Sangwoo Kim, Hye Ryun Kim, Byoung Chul Cho, Korean Lung Cancer Consortium (KLCC)

**Affiliations:** 1 Division of Medical Oncology, Department of Internal Medicine, Yonsei Cancer Center, Yonsei University College of Medicine, Seoul, Korea; 2 Severance Biomedical Science Institute, Yonsei University College of Medicine, Seoul, Korea; 3 Department of Pharmacology, Yonsei University College of Medicine, Seoul, Korea; 4 Department of Biomedical Systems Informatics, Brain Korea 21 PLUS Project for Medical Science, Yonsei University College of Medicine, Seoul, Korea; 5 Division of Hematology-Oncology, Department of Internal Medicine, Gachon Medical School, Gil Medical Center, Incheon, Korea; 6 Division of Hematology and Oncology, Department of Internal Medicine, Ulsan University Hospital, University of Ulsan College of Medicine, Ulsan, Korea; 7 Department of Oncology, Asan Medical Center, University of Ulsan College of Medicine, Seoul, Korea; 8 Division of Hematology-Oncology, Department of Medicine, Samsung Medical Center, Sungkyunkwan University School of Medicine, Seoul, Korea; 9 Department of Hematology-Oncology, Inje University Haeundae Paik Hospital, Busan, Korea; Mayo Clinic Rochester, UNITED STATES

## Abstract

The treatment of Lung adenocarcinoma (LUAD) could benefit from the incorporation of precision medicine. This study was to identify cancer-related genetic alterations by next generation sequencing (NGS) in resected LUAD samples from Korean patients and to determine their associations with clinical features. A total of 201 tumors and their matched peripheral blood samples were analyzed using targeted sequencing via the Illumina HiSeq 2500 platform of 242 genes with a median depth of coverage greater than 500X. One hundred ninety-two tumors were amenable to data analysis. *EGFR* was the most frequently mutated gene, occurring in 106 (55%) patients, followed by *TP53* (n = 67, 35%) and *KRAS* (n = 11, 6%). *EGFR* mutations were strongly increased in patients that were female and never-smokers. Smokers had a significantly higher tumor mutational burden (TMB) than never-smokers (average 4.84 non-synonymous mutations/megabase [mt/Mb] vs. 2.84 mt/Mb, *p* = 0.019). Somatic mutations of *APC*, *CTNNB1*, and *AMER1* in the WNT signaling pathway were highly associated with shortened disease-free survival (DFS) compared to others (median DFS of 89 vs. 27 months, *p* = 0.018). Patients with low TMB, annotated as less than 2 mt/Mb, had longer DFS than those with high TMB (*p* = 0.041). A higher frequency of *EGFR* mutations and a lower of *KRAS* mutations were observed in Korean LUAD patients. Profiles of 242 genes mapped in this study were compared with whole exome sequencing genetic profiles generated in The Cancer Genome Atlas Lung Adenocarcinoma. NGS-based diagnostics can provide clinically relevant information such as mutations or TMB from readily available formalin-fixed paraffin-embedded tissue.

## Introduction

Lung adenocarcinoma (LUAD) is the leading cause of cancer death worldwide. In particular, the incidence of LUAD is increasing in both never-smokers and females[1]. This means that prognosis and treatment of each patient can differ widely at the molecular level based on their gene expression patterns, copy number alterations, and mutations. Previous genomic studies of LUAD have shown that patients with driver gene mutations, such as those in epidermal growth factor receptor (*EGFR*) and anaplastic lymphoma kinase (*ALK*), receive a significant survival benefit from personalized therapy for LUAD [2, 3]. The recent discoveries of C-Ros oncogene 1, receptor tyrosine kinase *(ROS1)* and Ret proto-oncogene *(RET1)* fusions have raised expectations for the development of new targeted agents in LUAD. In molecularly selected patients, response rates to the appropriate targeted treatment can reach 60–70% or more, compared to the 20–30% response rate in an unselected population treated with conventional chemotherapy [4].

Ethnicity plays a distinct role in the prevalence of some genetic markers[5]. Asian patients with LUAD have a longer survival (11.0 vs. 8.9 months, *p* < 0.001), higher response rates (32.7 vs. 29.8%, *p* = 0.027), and greater toxicity in response to targeted therapy than Caucasian patients [6]. However, there is still a limited understanding of the genetic features of LUAD in Asian patients based on a lack of representation in existing public databases. Therefore, it is worthwhile to investigate whether these ethnic differences are due to genetic variation among ethnic groups. In this study, we investigated these variations in a Korean LUAD cohort. As we were able to sequence individual genomes, we examined these markers via next generation sequencing (NGS) technology, which can determine the profile of genetic changes in tumors, including single-nucleotide variations (SNVs), copy number variations (CNVs), and complex chromosomal rearrangements. NGS technology can provide a fast turnaround time and cost-effective sequencing for high numbers of targets. Given this, we sought to delineate a comprehensive characterization of the genomic landscape in Korean patients with LUAD using formalin-fixed paraffin-embedded (FFPE) surgical tissues and NGS technology. We have rendered to provide NGS results in a relevant time with simple FFPE samples rather than fresh tissue by targeted sequencing analysis, which is feasible to apply in clinical practice. Our data may serve as a reference in the development of precision medicine for Korean LUAD patients.

## Materials and methods

### Patients and data collection

A total of 201 LUAD patients with surgically resected primary lung cancer were prospectively enrolled from the Yonsei Cancer Center and Ulsan University Hospital between 2014 and 2016. All patients provided prior written informed consent, and this study was conducted with the approval of Institutional Review Board of Yonsei University Health System, Severance Hospital. A predesigned data collection format was used to review the patients’ electronic medical records for evaluation of clinicopathological characteristics and survival outcomes. Never-smokers were defined as those with a lifetime smoking dose of < 100 cigarettes. Ten tumor tissue sections (at least 10 μm thick) and patient blood samples (5 ml) were collected from prospectively recruited patients to differentiate between germline and somatic genetic aberrancy. Genetic analyses were performed in routine practice and included *EGFR* mutation and *ALK/ROS1* rearrangement. We uploaded raw NGS data to National Center for Biotechnology Information Sequence Read Archive (NCBI SRA) website for public access. (https://trace.ncbi.nlm.nih.gov/Traces/sra/, SRA accession ID is SRP200786.)

### Targeted sequencing of tumors

Genomic DNA was isolated from FFPE samples using the QIAamp DNA FFPE Tissue Kit (Qiagen, Hilden, Germany) for the targeted sequencing of 242 lung cancer-related genes selected based on a literature search (**S1 Table**) [2, 7, 8]. The genomic regions of the 242 genes were captured by the customized SureSelectXT Target Enrichment library generation kit (Agilent, Santa Clara, CA, USA) and sequenced on the Illumina HiSeq 2500 platform with a depth of coverage > 500X and a read length of 100 bp.

To do FFPE quality control and analysis, two cross validations were performed. First, we checked up and confirmed that FFPE precisely detect EGFR hotspot mutations which are the main target of LUAD therapy. We compared the NGS result with the PCR result regarding the EGFR hotspot mutations in the same sample. Another is to compare the results of the known frozen fresh (FF) data in public dataset. We first evaluated how similar the overall pattern of LUAD results of current study with that of The Cancer Genome Atlas (TCGA) dataset which was conducted by FF [2]. To evaluate the overall pattern of our data, we compared that of TCGA dataset [2]. This TCGA data set is composed of a total of 230 patients, of which the majorities (173 patients) were Caucasian [2].

### Variant calling and functional annotation

By default, base quality trimming for short reads from the targeted sequences was performed using Sickle[9]. Filtered reads were mapped to the human reference genome (GRCh37/hg19) using BWA[10]. All reads with a mapping quality score < 20 were discarded. The aligned reads (BAM file) were further processed with the Genome Analysis Tool Kit v3.5[11], including Mark Duplicate, Local Realignment, and Base Quality Score Recalibration. Candidates for somatic mutations were called by MuTect ver. 1.17[12] with default parameters. Somatic insertions/deletions were called by Scalpel [13] with default parameters. During somatic mutation calling, FoxoG sequencing artifacts [14] were removed using the Oxidative Damage Detection and Removal Tools (https://github.com/migbro/IGSB_oxoG_tools) to discard skewed read-orientation variants with the FoxoG parameter 0.625. Even after FoxoG filtration, nine samples had unexpectedly large numbers of mutations (Z-score of tumor mutational burden (TMB) > 1) and thus were excluded from further analysis under suspicion of potential damage to DNA. Somatic variants that passed all filters were considered high-confidence variants. CNVs were called using a CNV kit [15]. CNVs in genes were defined as follows: deletion, 0 copies; loss, 1 copy; gain, 3 copies; and amplification, ≥ 4 copies. The functional impacts of high confidence variants were annotated with ANNOVAR software[16], based on the consequences, predicted impacts, and reported allele frequencies in the population. In particular, non-rare variants (minor allele frequency > 0.05 in gnomAD database [17]) were discarded to remove non-pathogenic variants. Finally, CIVic and DoCM databases were used for clinical interpretation of variants in cancer. TMB was measured by the number of non-synonymous missense mutations per megabase (Mb) within the range of the targeted capture region. An ‘Oncoprint’ is a way to visualize overall genomic alteration events using a heatmap. Mutations of each sample on the Oncoprint are aligned in a mutually exclusive manner. For example, the samples with the highest frequency in the entire sample are aligned on the top left, and the samples with the next highest frequency are aligned on the back. This is a kind of clustering that can easily distinguish between co-occurrence and mutually exclusive patterns on the major genes. It was drawn using the bioconductor package ‘Complex Heatmap’ [18] in R ver. 3.4. Using the package ‘maftools’ [19], lollipop plots were drawn for frequently mutated genes to check the recurrence of genomic loci with variants, and somatic interactions between mutually exclusive or co-occurring sets of genes were investigated. Mutations and putative CNVs stored in cBioportal[20, 21] were used for the above genomic analysis. Pathway diagrams were depicted using Pathway Mapper[22]. To identify the clinical importance of mutations, we created a mutation classification system based on knowledgebase databases and a computational prediction algorithm. Clinical importance was ranked using CIVic(criteria: predictive & sensitive & evidence level = {A, B, C} & supports categories), Cancer Genome Interpreter (CGI) (criteria: drug prescription & responsive & alteration match–complete categories)[23], and CRAVAT(criteria: CHASM FDR ≤ 0.1 & TARGET DB only categories), sequentially. To confirm how many ranked mutations were included, Venn diagrams were drawn using Venny [24].

### Statistical methods

All statistical analyses were performed using R and Python (Scipy and Seaborn packages). Student's *t-*test or Fisher's exact test was used for group comparisons. Disease-free survival (DFS) was measured from the date of diagnosis to tumor recurrence or death, while overall survival (OS) was measured from the date of diagnosis until the date of death. Patients were censored on October 2017 if alive and recurrence free. Patients without a known date of death were censored at the time of last follow-up. A log rank test for mutations of each gene, signaling pathways, and TMB was used to compare the DFS between groups. Two-sided *p*-values < 0.05 were considered significant.

## Results

### Clinical characteristics

We enrolled 201 patients with LUAD, and their characteristics are summarized in **Tables 1 and S2.** This entire cohort included 87 men and 114 women; the median age was 63 years (range, 34–83), and 157 patients (78.1%) had stage I or II disease at initial diagnosis. One hundred twenty-five patients (62.2%) were never-smokers; never-smokers were defined as those with a lifetime smoking dose of < 100 cigarettes. One hundred nineteen patients (59.2%) were urban residents and 68 patients (33.8%) were non-urban residents. Ninety-four patients (46.8%) had adjuvant platinum-based chemotherapy as a standard treatment. *EGFR* mutations (51.8%, 57/110), *ALK* rearrangements (4.1%, 4/98), and *ROS1* rearrangements (0.7%, 1/140) were identified based on genetic mutation testing in routine practice. The mean follow-up period was 42 months (range, 7–114 months). During follow-up, recurrence was observed in 55 patients (27.4%), and median DFS was 89 months (95% confidence interval [CI], range 63.68–114.32 months). DFS was separated by each stage (**S1 Fig**). Since only 10 patients died, the median OS was not yet reached (**Table 2**).

**Table 1:** Baseline patient characteristics of the analytic population.

Characteristic	Median (Range)	Analytic Population (N = 201)
**Age in years**	63 (34–83)	
**Sex**		
**Male**		87 (43.3%)
**Female**		114 (56.7%)
**Smoking**		
**Never smoker**		125 (62.2%)
**Former smoker**		51 (25.4%)
**Current smoker**		25 (12.4%)
**Residence**		
**Urban**		119 (59.2%)
**Non-urban**		68 (33.8%)
**Unknown**		14 (7.0%)
**ECOG**		
**ECOG 0, 1**		199 (99.0%)
**ECOG 2**		2 (1%)
**Stage**		
**IA, IB**		113 (56.2%)
**IIA, IIB**		44 (21.9%)
**IIIA, IIIB, IIIC**		42 (20.9%)
**IV**		2 (1.0%)
**EGFR Mutation (N = 110)**^a)^		57 (51.8%)
**Exon 19 del, L858R**		42 (73.7%)
**Others**^**b)**^		15 (26.3%)
**ALK Fusion (N = 98)**^**c)**^		4 (4.1%)
**ROS1 Rearrangement (N = 140)**^**d)**^		1 (0.7%)
**Adjuvant chemotherapy**		
**Yes (Platinum doublet)**		94 (46.8%)
**Yes (Others)**^**e)**^		1 (0.05%)
**No**		106 (52.7%)

a) EGFR mutation test was performed using the Peptide nucleic acid (PNA) clamping method in 110 patients.

b) L861Q, T790M, exon20 insertion: c.2316_2317ins TACAACCCC; exon20 mutation: Ser768Ile, c.2303G>T, Val774Met, c.2320G>A; exon21 mutation: Leu858Arg, c.2573T>G, G719X, S768I

c) ALK fusion test was performed by fluorescence *in situ* hybridization (FISH) analysis in 98 patients.

d) ROS1 rearrangement test was performed by immunohistochemistry (IHC) analysis in 140 patients.

e) One patient enrolled adjuvant erlotinib clinical trial.

**Table 2:** Clinical outcomes of the analytic population.

Characteristic	Median (Range)	Analytic Population (N = 201)
**Recurrence**		
**Yes**		55 (27.4%)
**No**		146 (72.6%)
**Death**		10 (0.05%)
**Disease free survival (DFS)**	89 months (63.68–114.32)	
**Overall survival (OS)**	Median not reached	

### Genomic landscape of LUAD

We analyzed 192 of 201 samples, after excluding nine with excessive FoxoG artifacts. To confirm FFPE quality control, we compared the NGS result with their PCR result on the well-known EGFR hotspot mutations (i.e.S768I, L858R, L861Q, E19 DEL) and identified that about 90% (45/51) were identical to each other. Next, we evaluated the overall pattern of our data compared with that of TCGA data set as positive control (**S2 Fig**) [2]. We confirmed that the mutation patterns of *EGFR*, *TP53*, *KRAS*, and *PIK3CA* of our data are the comparable with TCGA (**S2A Fig**). In TCGA LUAD dataset, *EGFR* hotspot mutations were observed in L858R, exon19 deletion and other hotspot mutations order, which is similar pattern to our result (**S2B Fig**). *TP53* tends to be widely distributed in the DNA binding domain (**S2C Fig**). *KRAS* (G12, Q61) and *PIK3CA* (E542, E545, H1047) were observed in TCGA dataset, which are comparable with ours (**S2D** and **S2E Fig**). In terms of CNV, we found out that the oncogene and tumor suppressor gene (TSG) are similar to TCGA and COSMIC (https://cancer.sanger.ac.uk/cosmic) (**S2F Fig**). Collectively, based on the quality control process, we analyzed genetic analysis in this study.

**Fig 1:**
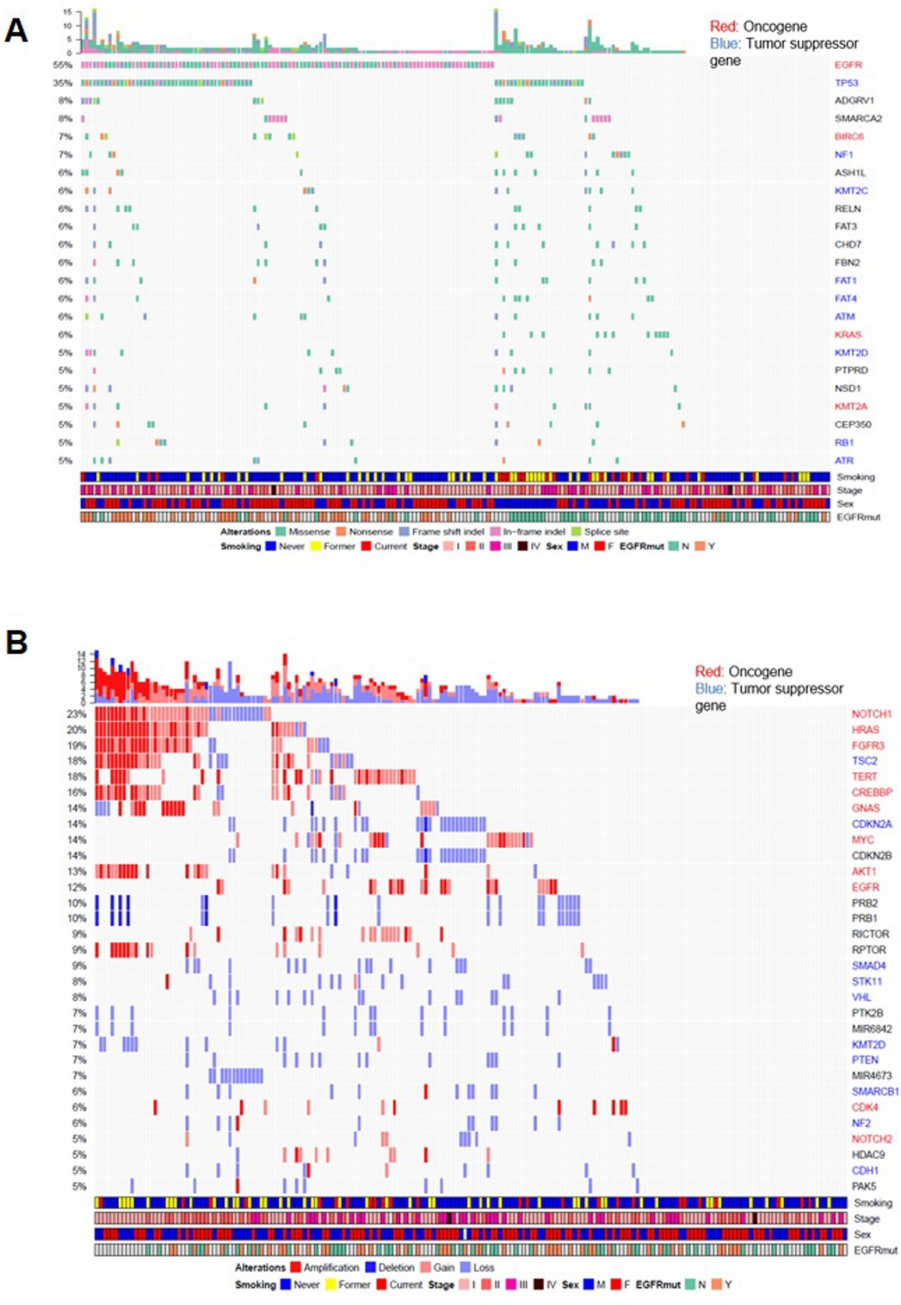
Genomic landscape of lung adenocarcinoma (LUAD). **(A)** This is the oncoprint of the somatic single-nucleotide variations (SNVs) in 192 LUAD patients. SNV alterations included missense, nonsense, frameshift indel, in-frame indel, and splice site mutations. (Red, oncogene; Blue, tumor suppressor gene) The upper bar chart is the total number of mutations or CNVs. The order of the genes is performed by the frequency of the mutations or CNVs across all samples. **(B)** This is the oncoprint of the somatic copy number variations (CNVs) in 192 LUAD patients. CNV alterations included copy number gain, amplification, loss, or deletion.

A total of 761 somatic non-silent SNVs and 388 insertions and deletions (indels) were identified from the targeted sequencing regions of the 192 tumors, corresponding to a median of 2.08 SNVs per Mb. The Oncoprint demonstrated that SNV alterations included missense, nonsense, frameshift indel, in-frame indel, and splice site mutations. We found that *EGFR* was the most frequently mutated gene (n = 106, 55%), followed by *TP53* (n = 67, 35%) and *KRAS* (n = 11, 6%) **(Fig 1A)**. *EGFR* and *KRAS* mutations were mutually exclusive **(S3A Fig)**. *EGFR* mutation were strongly enriched in patients that were female and never-smokers. In female and never-smokers, between patients with *EGFR* mutation and *EGFR* wild type are statistically significant (*p* = 0.005). Rates of mutations in other genes were 8% for *ADGRV1* (n = 15), 8% for *SMARCA2* (n = 15), 7% for *BIRC6* (n = 13), 7% for *NF1* (n = 13), 6% for *RELN* (n = 12), 6% for *KMT2C* (n = 12), 6% for *FAT3* (n = 12), 6% for *ATM* (n = 11), and 5% for *RB1* (n = 9) **(Fig 1A)**. Copy number gain or amplification was detected in *HRAS* (n = 38, 20%), *FGFR3* (n = 36, 19%), *TERT* (n = 34, 18%), *CREBBP* (n = 30, 16%), *MYC* (n = 26, 14%), *AKT* (n = 25, 13%), and *EGFR* (n = 24, 12%). Copy number loss or deletion was observed in *CDKN2A* (n = 26, 14%), *SMAD4* (n = 17, 9%), *VHL* (n = 15, 8%), *STK11* (n = 16, 8%), *PTEN* (n = 13, 7%), and *KMT2D* (n = 13, 7%) **(Fig 1B).** We found that smokers had a significantly higher TMB than never-smokers (average 4.84 vs. 2.84 mt/Mb, respectively, *p* = 0.019) **(S3B Fig)**.

### Mutation mapper plot and pathway diagram

**Fig 2:**
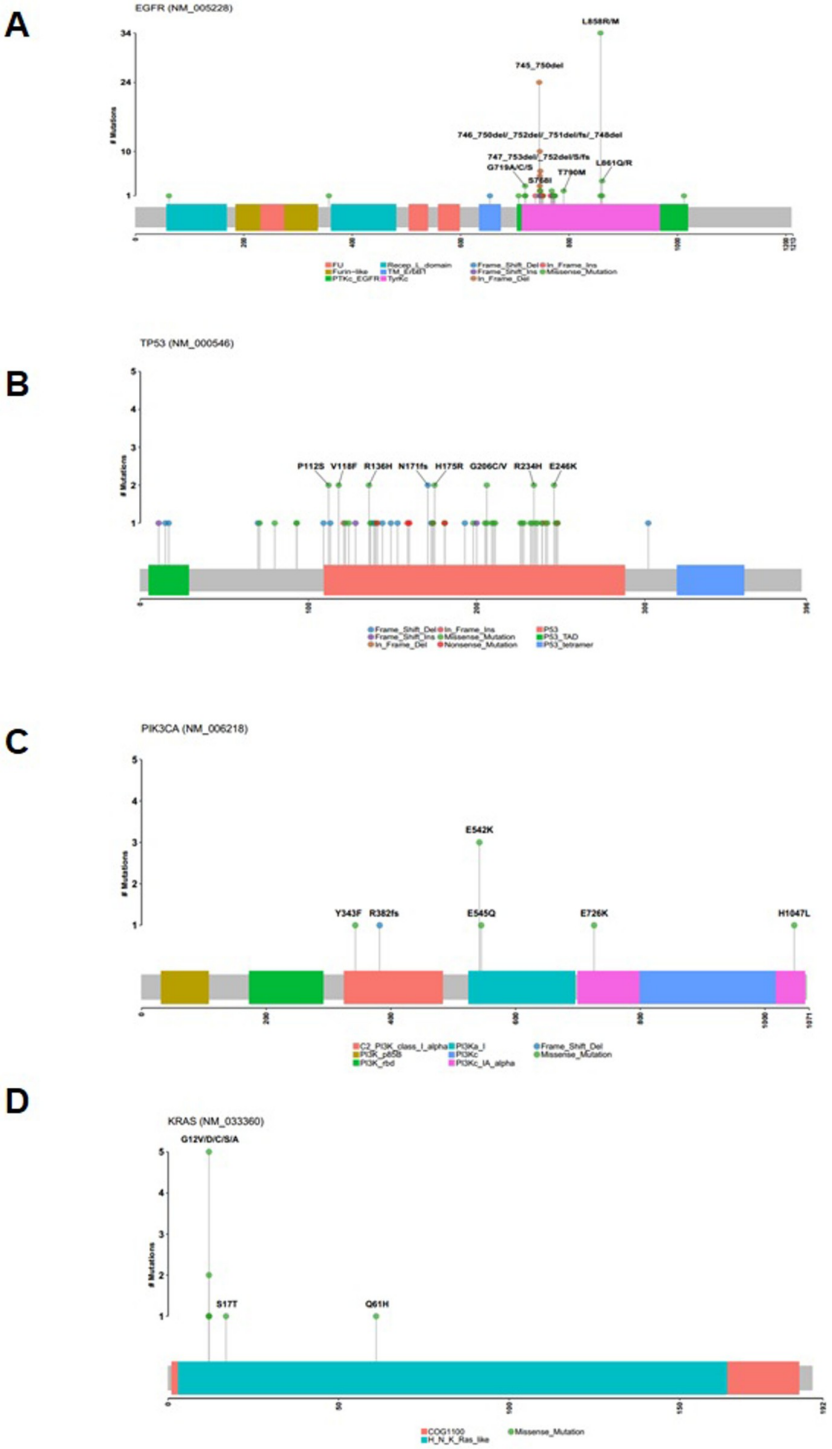
Gene mutation mapper plot of *EGFR*, *TP53*, *PIK3CA*, and *KRAS*. **(A)** Among *EGFR* mutations, L858R/M and exon 19 deletion were the most common alterations, followed by L861Q/R. **(B)** In *TP53*, P112S, V118F, R136H, N171fs, H175R, G206C/V, R234H, and E246K mutations were identified at similar rates. **(C)** In *PIK3CA*, the established canonical E542K missense mutation was the most common. **(D)** Among *KRAS* mutations in codon 12, G12V/D/C/S/A was the most common.

In the mapper plot for *EGFR*, L858R and exon 19 deletion were the most common alterations, observed in 35 samples (18%) and 55 samples (29%), respectively. This was followed by L861Q, observed in 5 samples (3%). In the mapper plot for *TP53*, the P112S (n = 2, 1%), V118F (n = 2, 1%), R136H (n = 2, 1%), N171fs (n = 2, 1%), H175R (n = 2, 1%), G206C/V (n = 2, 1%), R234H (n = 2, 1%), and E246K (n = 2, 1%) mutations were identified at similar rates. In *PIK3CA*, the established canonical E542K missense mutation was the most common (n = 3, 2%). In addition, among *KRAS* mutations in codon 12, G12D/V/C/S/A was the most common (n = 10, 5%) **(Fig 2).** We also depicted pathway diagrams of four canonical pathways: canonical WNT, cell cycle, PI3K, and RTK-RAS **(S4 Fig)** [25]. In the canonical WNT pathway, we observed rates of 3% for SNVs in *CTNNB1* (n = 6, 5 missense mutations, 1 nonsense mutation), 4% for SNVs in *APC* (n = 8, 2 missense mutations, 2 nonsense mutations, 3 frameshift indels, 1 splice site mutation), 1% for SNVs in *AMER1* (n = 1 missense mutation), and 1% for CNVs in *APC* (n = 2, 1 gains with 3 copies, 1 loss with 1 copy) (**S4A Fig**).

### Clinical implication with somatic mutation classification system for LUAD

**Fig 3:**
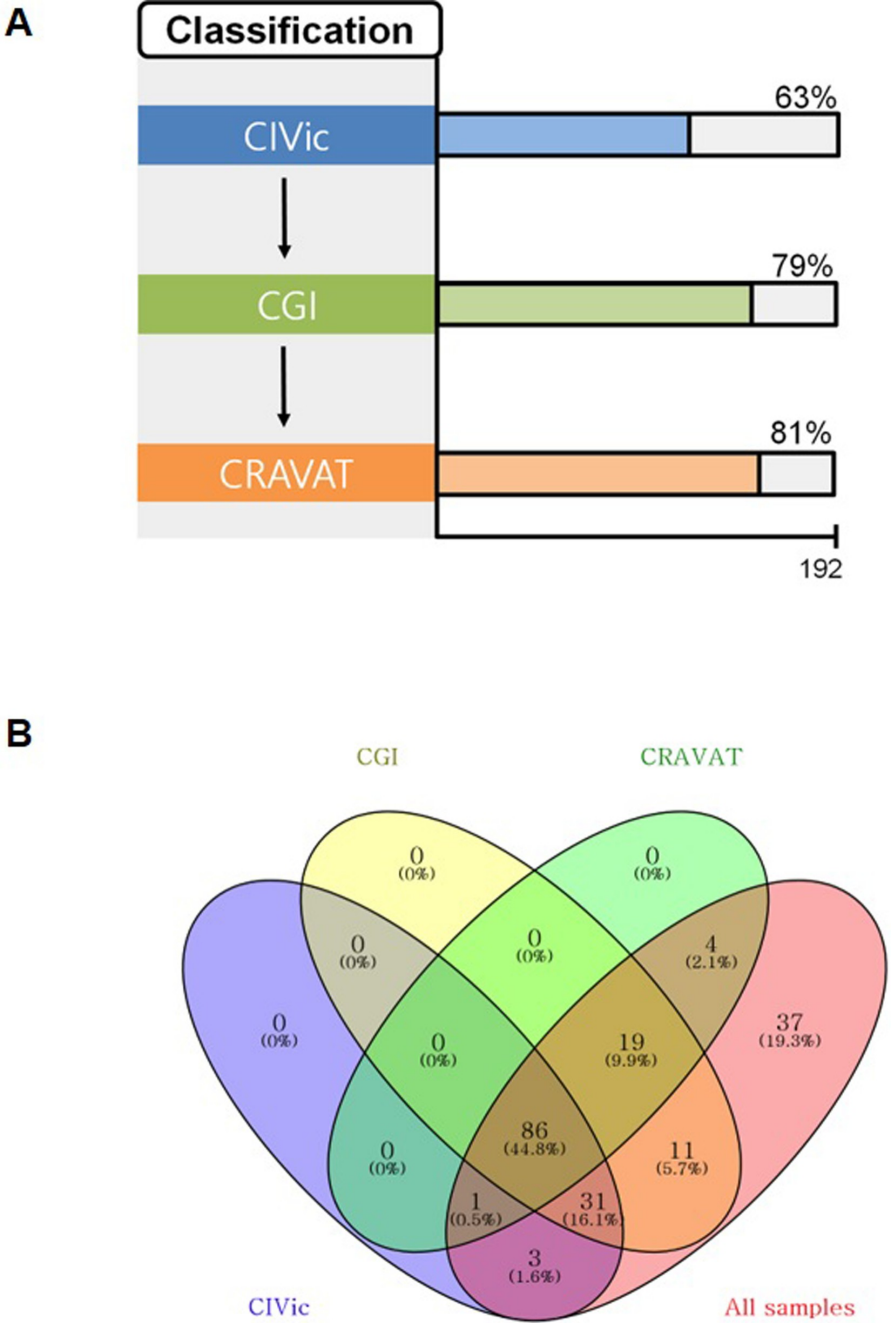
Mutation classification system. **(A)** Hierarchical mutation classification system based on knowledgebase database and the computational prediction algorithm. **(B)** Venn diagram to confirm how many ranked mutations were included.

We attempted to implement a precision medicine approach for application in the clinical field. The purpose of precision medicine through NGS is to determine the link between each mutation with an associated targeted therapy and the clinical outcome in cancer patients. Although there are many clinical annotation databases for various somatic mutations, the determination of which mutations have clinical implications differs slightly in each. Hence, a harmonized system for a meta-knowledgebase of clinical interpretations of cancer genomic variants is required to reliably determine clinical implications for as many patients as possible[26]. Of 192 LUAD patient samples, 121 samples (63%) were clinically annotated in CIVic[27], validated through various publications and clinical trials, and annotated through CGI[23], resulting in the annotation of 151 samples (79%). Potential targets that still remain are annotated with CRAVAT[28] (155 samples, 81%), which involves computational prediction **(Fig 3A)**. There were a total of 86 samples annotated in the three databases, 3 of which were annotated only in CIVic, 11 only in CGI, and 4 only in CRAVAT **(Fig 3B)**. The somatic mutations reported in CIVic were the well-known *EGFR* L858R, exon 19 deletion, and T790M mutations; the G12V/D/C/S/A mutation in *KRAS*; E542K in *PIK3CA*; and Y220C and R175H in *TP53*. Genes with CNVs included *CDKN2A*, *EGFR*, and *PTEN*, among others. Somatic mutations annotated only in CGI were *ARID1A*, *BRAF*, *BRCA*, *STK11*, and *BAP1*, while *SETD2* and *STK11* were the annotated somatic CNVs. The somatic mutations independently estimated by CRAVAT were H179R and G245C for *TP53* and P750R for *DNMT3A*. Prospective application of this approach should be assessed in a future umbrella trial of lung cancer patients.

### Clinical correlation

**Fig 4:**
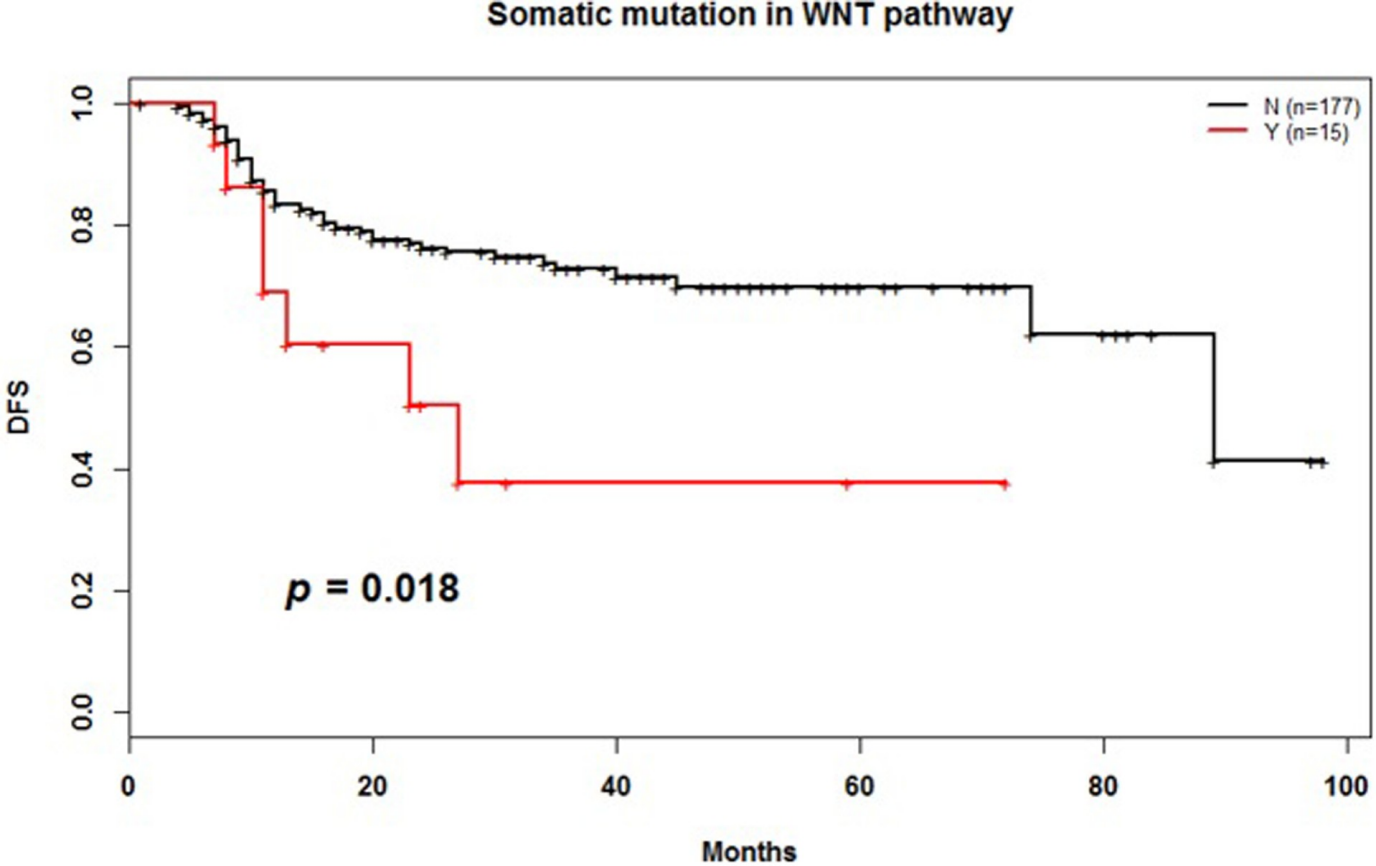
Disease-free survival (DFS) by somatic mutation in the canonical WNT pathway (*p* = 0.018). *APC*, *CTNNB1*, and *AMER1* were mutated in the WNT pathway.

**Fig 5:**
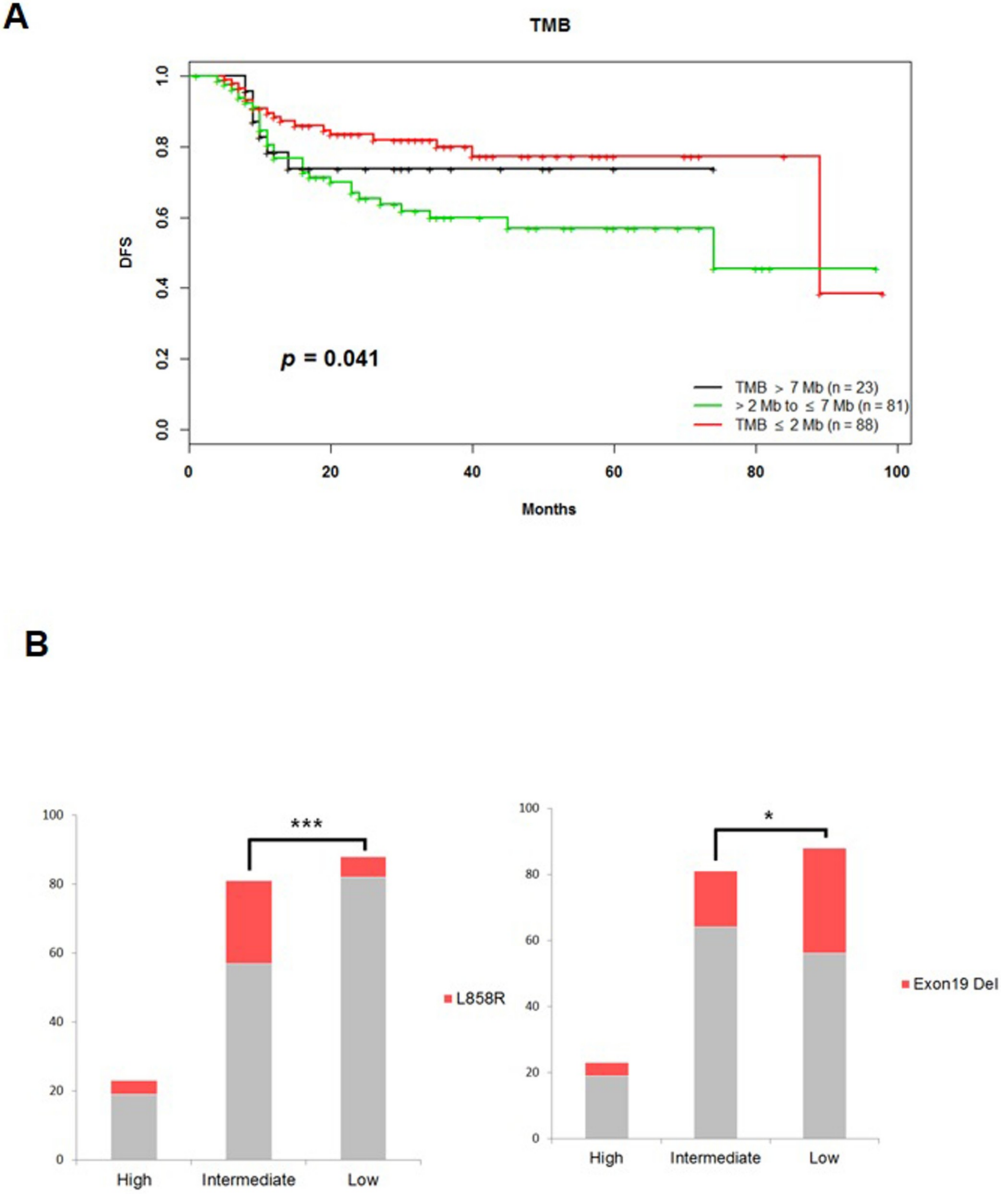
Disease-free survival (DFS) by tumor mutation burden (TMB). **(A)** Kaplan-Meier plots showing prognostic effect of nonsynonymous TMB on disease-free survival (*p* = 0.041). **(B)** The low TMB group had more *EGFR* exon 19 deletions among various mutations than other groups (36%). In the intermediate TMB group, *EGFR* L858R was the most common (30%). Fisher’s exact test was used to compare low and intermediate TMB groups for the *EGFR* exon 19 deletion (*p* = 0.041) and *EGFR* L858R variants (*p* < 0.001). No significant differences were found for other comparisons.

Among 192 patients with available NGS and survival data, *TP53* (*p* = 0.062), *EGFR* (*p* = 0.299), the RTK-RAS pathway (*p* = 0.089), and the PI3K pathway (*p* = 0.149) were not associated with shorter DFS **(S5 Fig)**; however, mutations in *APC* (8 samples with 2 missense mutations, 2 nonsense mutations, 3 frame shift indels, and 1 splice site mutation; 2 samples with 1 gain and 1 loss), *CTNNB1* (6 samples with 5 missense mutations and 1 nonsense mutation), and *AMER1* (1 sample with 1 missense mutation) in the canonical WNT pathway were associated with shorter DFS (*p* = 0.018) (**Fig 4**). In addition, based on TMB annotations, cut-offs were used to divide patients into tertiles of low (≤ 2 mt/Mb, n = 88), intermediate (> 2 to ≤ 7 mt/Mb, n = 81), and high TMB (> 7 mt/Mb, n = 23) groups, and these were associated with differences in DFS. Patients with a low TMB showed better prognosis than those with high or intermediate TMB (*p* = 0.041) (**Fig 5A**). The low TMB group had more *EGFR* exon 19 deletions than the other groups (36%). In the intermediate TMB group, *EGFR* L858R was the most common mutation (30%) (**Fig 5B**).

## Discussion

Our study shows that it is feasible to incorporate NGS into the clinical care of lung cancer patients. Through our NGS analysis, the most common genomic alterations (*EGFR*, *TP53*, *ADGRV1*, and *SMARCA2*) were slightly different from those observed in present investigations of LUAD in Caucasian [2]. In The Cancer Genome Atlas (TCGA), the rate of *KRAS* mutation in LUAD is 33%, while that of *EGFR* mutation is only 14%[2]. It should be noted that we have a higher proportion of female (56.2%) and non-smokers (62.2%) than is found in TCGA. However, the most prominent difference is the ethnicity of the patients. Only eight Asian patients are included in TCGA[2]. We analyzed only ethnic Korean patient samples and can conclude that *EGFR* mutation is the most common (55%) in Koreans, based on the current study and a rate of 59% among Asian patients with LUAD in previous reports [29]. Since *KRAS* mutation occurs exclusive of *EGFR* mutation, *KRAS* mutations are slightly less frequent in ethnic Koreans than in Caucasian patients. Luo published the results of whole genome sequencing for young never-smoked Asian with lung adenocarcinoma[30]. Compared with this study, we conducted in a more practical way by targeted sequencing with FFPE. In Luo study, *EGFR* mutation was found to be somatic SNV 25% and CNV 19% but ours was 64%, 15% respectively. And *KRAS* of Luo study was sSNV 11% but ours was 2%. Interestingly, several genes showed almost the same ratio (*TP53* sSNV, Luo 28% vs. ours 31%; *MYC* sCNV, 14% vs. 10%; *TERT* sCNV, 17% vs. 17%, respectively).

Similar to the discovery of *EGFR*, *ALK*, and *ROS1*, various studies for identifying molecular characterizations of LUAD are under way, and our study is also part of this effort. Mutations in specific genes affect not only the carcinogenic process but also dysfunction of signaling pathways and can be important mediators in tumorigenesis[31]. The WNT pathway is involved in the formation of lung homeostasis and tumor angiogenesis[32]. WNT pathway aberrations are potential therapeutic targets in lung cancer patients[33]. The most studied WNT pathway mutations in cancers include sporadic mutations in *APC* and β-catenin genes. Since *APC* is part of the degradation scaffold for β-catenin, mutations of *APC* result in reduced degradation and increased nuclear accumulation of β-catenin, leading to activation of target oncogenes including cyclin D1 and c-Myc[33]. Clinical trials of WNT signaling pathway inhibitors have been conducted in advanced solid tumors (NCT03355066). Our analysis also shows that patients with *APC*, *CTNNB1*, and *AMER1* mutations in the WNT pathway show shorter DFS compared to wild-type patients (**Fig 4**). In addition, we investigated the clinical significance of TMB in patients with LUAD and examined the relationship between TMB and prognosis. TMB is thought to be associated with the amount of tumor neoantigen and to have an important role in predicting the effect of immune checkpoint inhibitors[34]. We found that smokers had a significantly higher TMB than never-smokers (average 4.84 vs. 2.84 mt/Mb, respectively, *p* = 0.019). Devarakonda et al. also annotated a TMB greater than 8 mt/Mb as high and reported a better prognosis in this group[35]. On the other hand, Owada-Ozaki reported that shorter OS and DFS was associated with high TMB in stage I NSCLC[34]. In our data, patients with a TMB < 2 mt/Mb showed longer DFS than patients with a TMB ≥ 7 mt/Mb (*p* = 0.041) (**Fig 5A**). Since there are still many conflicting results, further studies are needed to validate TMB as a prognostic marker. Notably, exon 19 deletion was the most common mutation in the low TMB group, which exhibited good prognosis. It is already known that exon 19 deletion results in a better prognosis than other *EGFR* mutations[36] **(Fig 5B).**

In order for the above analyses to be applied to clinical practice, appropriate use of a meta-knowledgebase of clinical implications of cancer genomic variants is necessary[37]. A meta-knowledge-based framework of holistic interpretation comprehensively covers hundreds of genes, disease and drugs. Hence, we included predicted target mutations in CRAVAT, as well as providing annotations via CIVic and CGI. Overall, this methodology may expedite the widespread implementation of an umbrella trial of lung cancer patients.

Several technical limitations were identified in this study. First, a low tumor cellularity in samples, owing to normal cell contaminants, and high levels of intra-tumor heterogeneity make it difficult to accurately call SNVs and CNVs. For this reason, the variant allele frequency was lower than the theoretical value of 0.5 (**S6 Fig**). Second, targeted sequencing for the identification of CNVs remains a secondary option when more sensitive methods, such as whole-genome sequencing or specialized array-based methods, are unavailable. As targeted sequencing-based CNV analysis generally performs better in a larger cohort, the size and sustainability of clinical trials should be considered when they are designed. Third, the NGS platform used in this study detected only SNVs and CNVs although diverse structural variations and epigenetic events exist outside of the captured exons. Active participation of genome analysis experts is strongly recommended to manage these technical issues. Finally, since we used only 242 genes in this study, other factors including genetic alteration in other genes, epigenetic alterations, gene and protein expression may be related to LUAD risk. There are recent reports that exposure to outdoor particulate matter (PM_10_)[38, 39] or indoor secondhand smoke and high temperature cooking oil fumes[40] are associated with lung cancer. Since, there were inadequate information for patient’s dwelling or occupation, it was precluded to analyze environmental factor.

## Conclusions

In conclusion, targeted sequencing using NGS can provide clinically relevant mutation profiling information from readily available FFPE tissues. *EGFR* was the most frequently mutated gene (55%), followed by *TP53* (35%) and *KRAS* (6%). This may assist in decision to the use of innovative clinical trials of genotype-matched drugs and provide benefits to many cancer patients.

## Supporting information

S1 Fig Disease-free survival (DFS) by stage in lung adenocarcinoma (LUAD) patients (*p* < 0.001). (TIF)

S2 Fig Comparison of genetic alteration pattern between current study and TCGA data. **(A)** Comparison of SNV between current study and TCGA data. **(B)** Comparison of EGFR hot spot mutation between current study and TCGA data. **(C)** Comparison of TP53 hot spot mutation between current study and TCGA data. **(D)** Comparison of KRAS hot spot mutation between current study and TCGA data. **(E)** Comparison of PIK3CA hot spot mutation between current study and TCGA data. **(F)** Comparison of CNV between current study and TCGA data.
(PDF)

S3 Fig Co-occurrence and tumor mutation burden (TMB) test. **(A)** *EGFR* and *KRAS* mutations were mutually exclusive. **(B)** Smokers had a significantly higher TMB than never-smokers (average 4.84/Mb vs. 2.84/Mb, respectively, *p* = 0.019).
(PDF)

S4 Fig Pathway diagrams. **(A)** Pathway mapper diagrams of canonical WNT signaling, **(B)** cell cycle, **(C)** PI3K, and **(D)** RTK-RAS pathways. (Red, oncogene; Blue, tumor suppressor gene).
(PDF)

S5 Fig **Disease-free survival (DFS) by somatic mutations** in **(A)** *TP53*, **(B)** *EGFR*, **(C)** RTK-RAS pathway, and **(D)** PI3K pathway.
(PDF)

S6 Fig Variant allele frequency (VAF). (TIF)

S1 Table A list of 242 genes and information of genomic location used for targeted sequencing. (XLS)

S2 Table Individual sample lists including detailed clinical information. (XLSX)
