# Supplementary figures and images for: Molecular characterization of lung adenocarcinoma from Korean patients using next generation sequencing
Source: PLoS One. 2019 Nov 25;14(11):e0224379. doi: 10.1371/journal.pone.0224379 (PMC6876835; doi:10.1371/journal.pone.0224379)

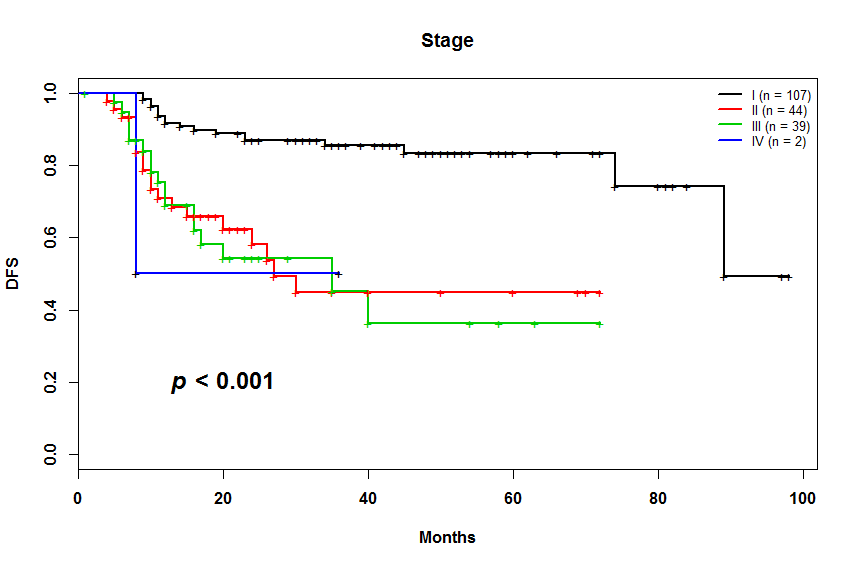

Supplement: S1 Fig — (TIF) [file pone.0224379.s001.tif]

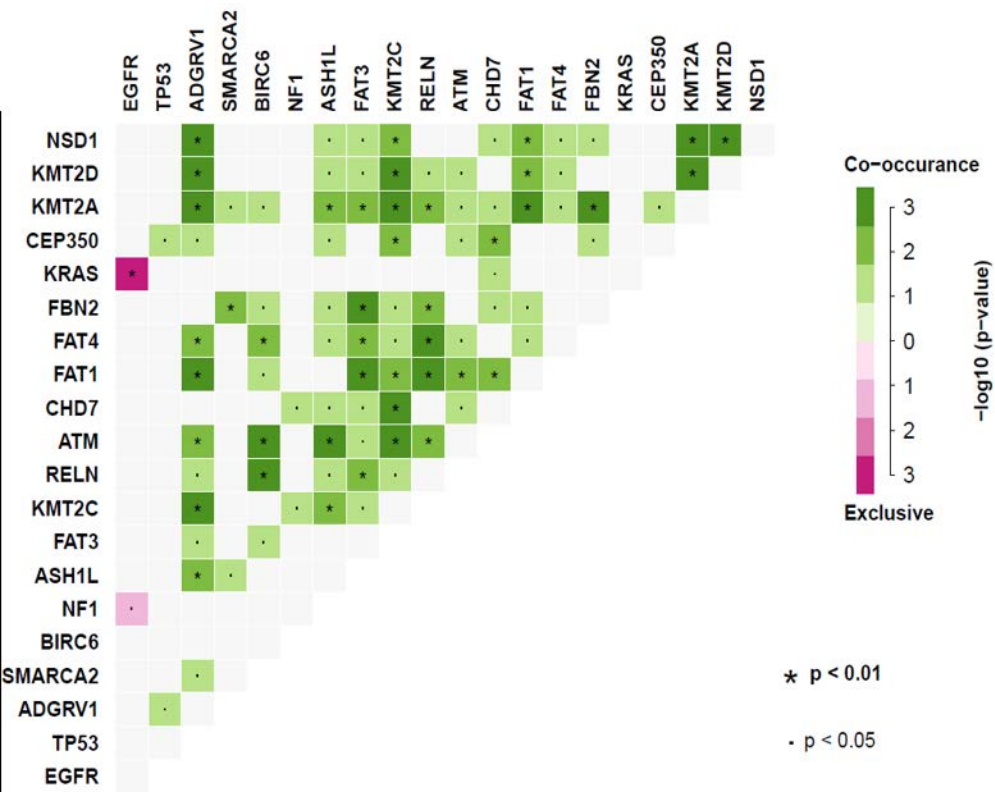

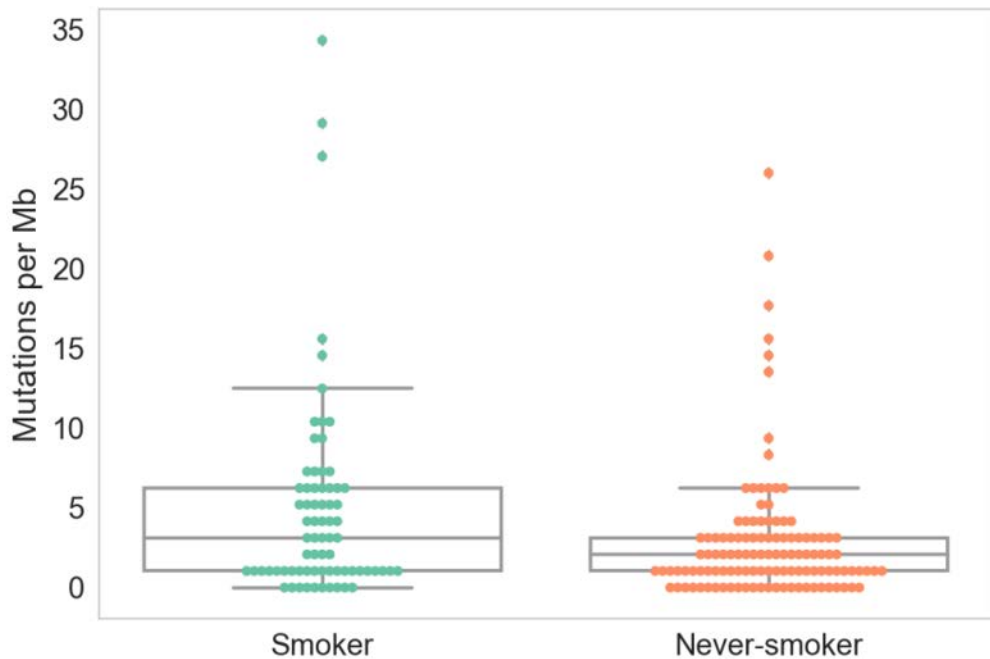

Supplement: S3 Fig — (A) EGFR and KRAS mutations were mutually exclusive. (B) Smokers had a significantly higher TMB than never-smokers (average 4.84/Mb vs. 2.84/Mb, respectively, p = 0.019). (PDF) [file pone.0224379.s003.pdf]

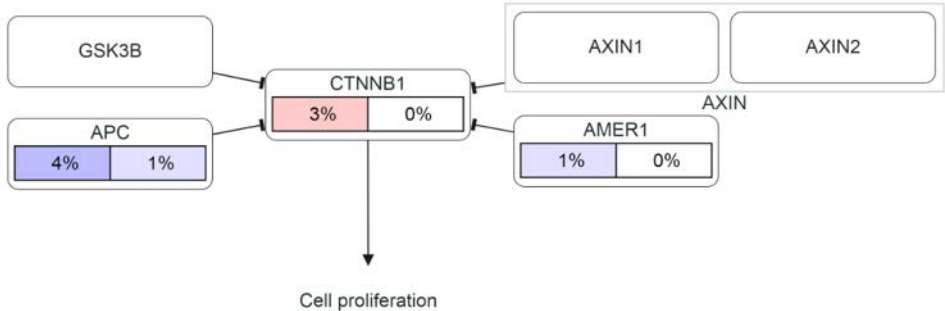

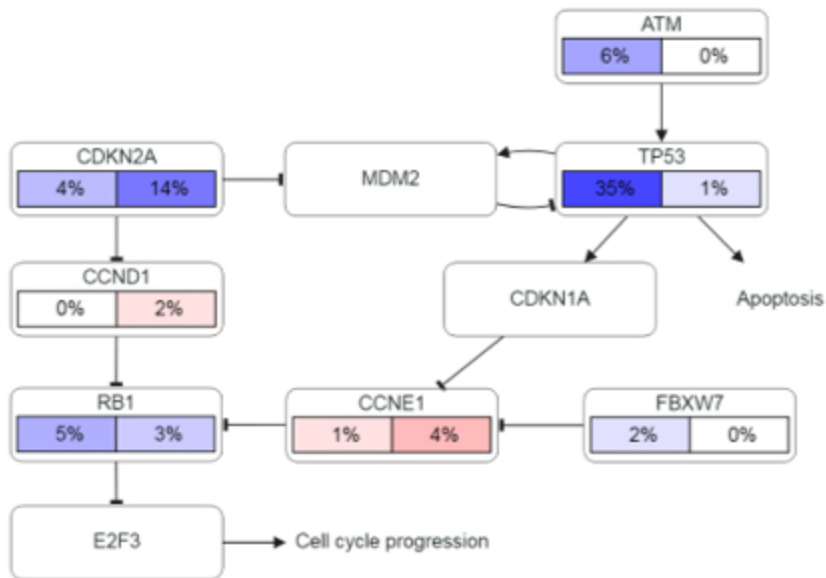

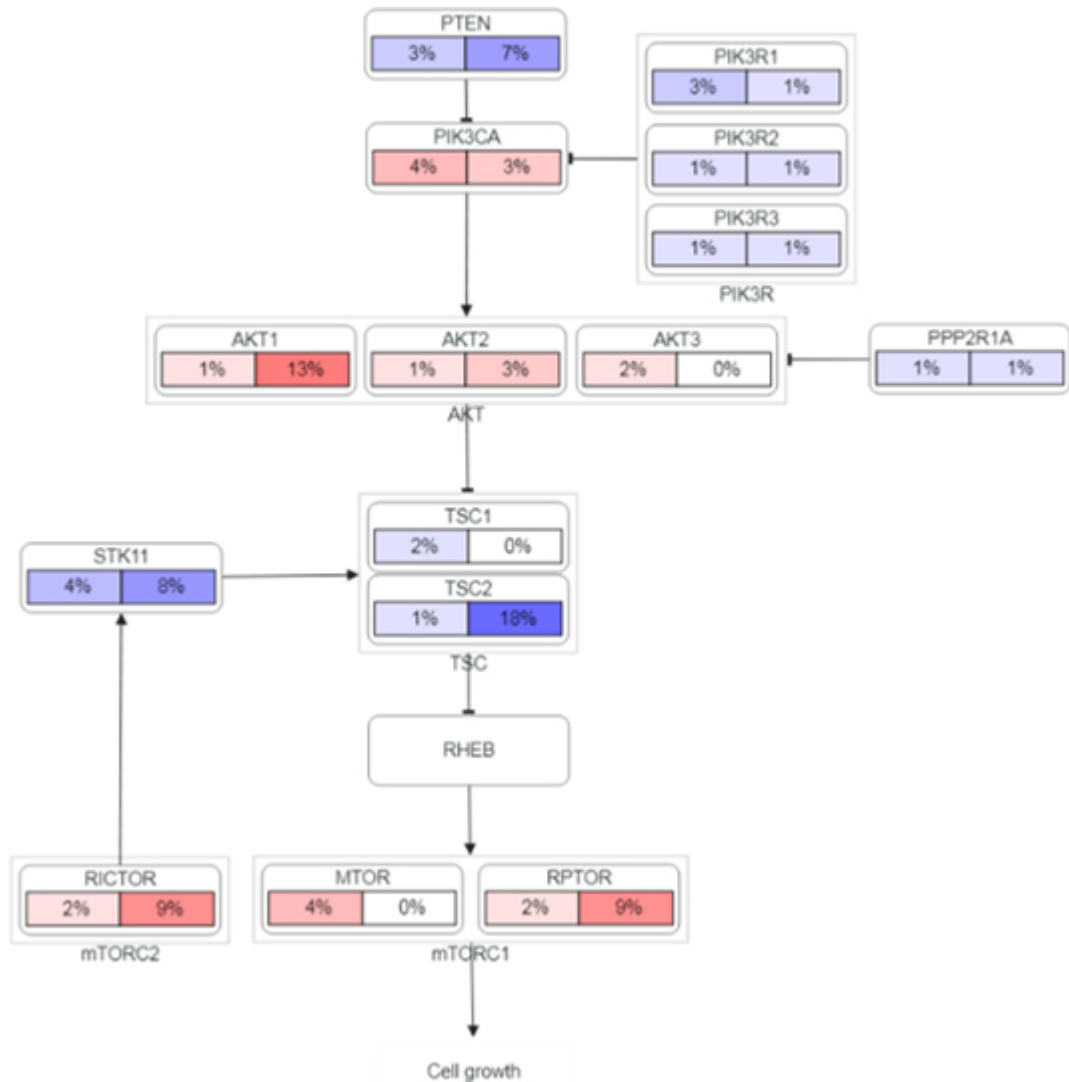

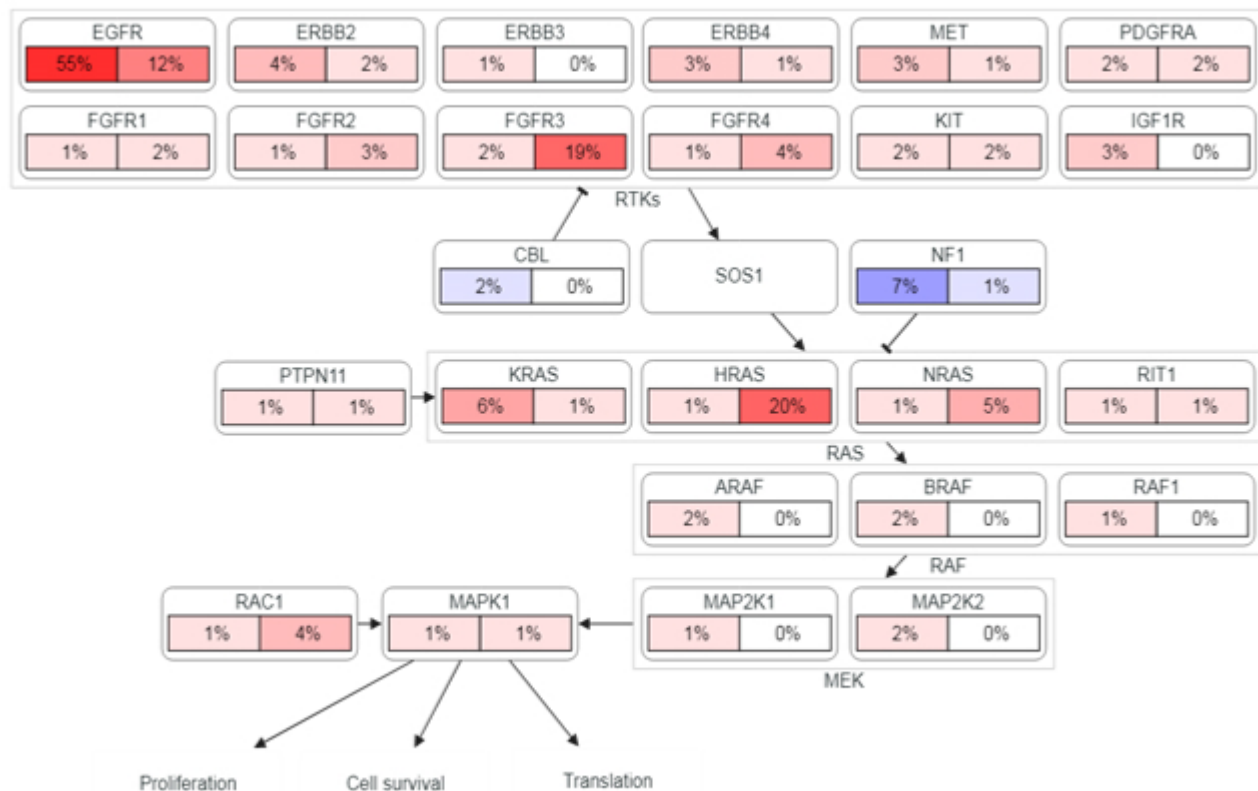

Supplement: S4 Fig — (A) Pathway mapper diagrams of canonical WNT signaling, (B) cell cycle, (C) PI3K, and (D) RTK-RAS pathways. (Red, oncogene; Blue, tumor suppressor gene). (PDF) [file pone.0224379.s004.pdf]

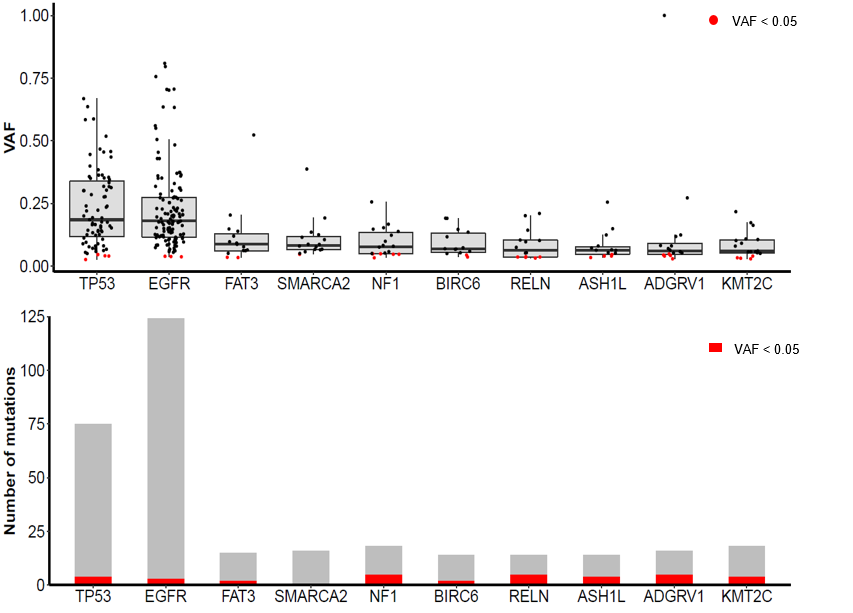

Supplement: S6 Fig — (TIF) [file pone.0224379.s006.tif]
