# Supplementary material for: Molecular characterization of lung adenocarcinoma from Korean patients using next generation sequencing
Source: PLoS One. 2019 Nov 25;14(11):e0224379. doi: 10.1371/journal.pone.0224379 (PMC6876835; doi:10.1371/journal.pone.0224379)

S2AFig. Comparison of *SNV* between current study and TCGA data

red: oncogene
blue: tumor suppressor gene

TCGA

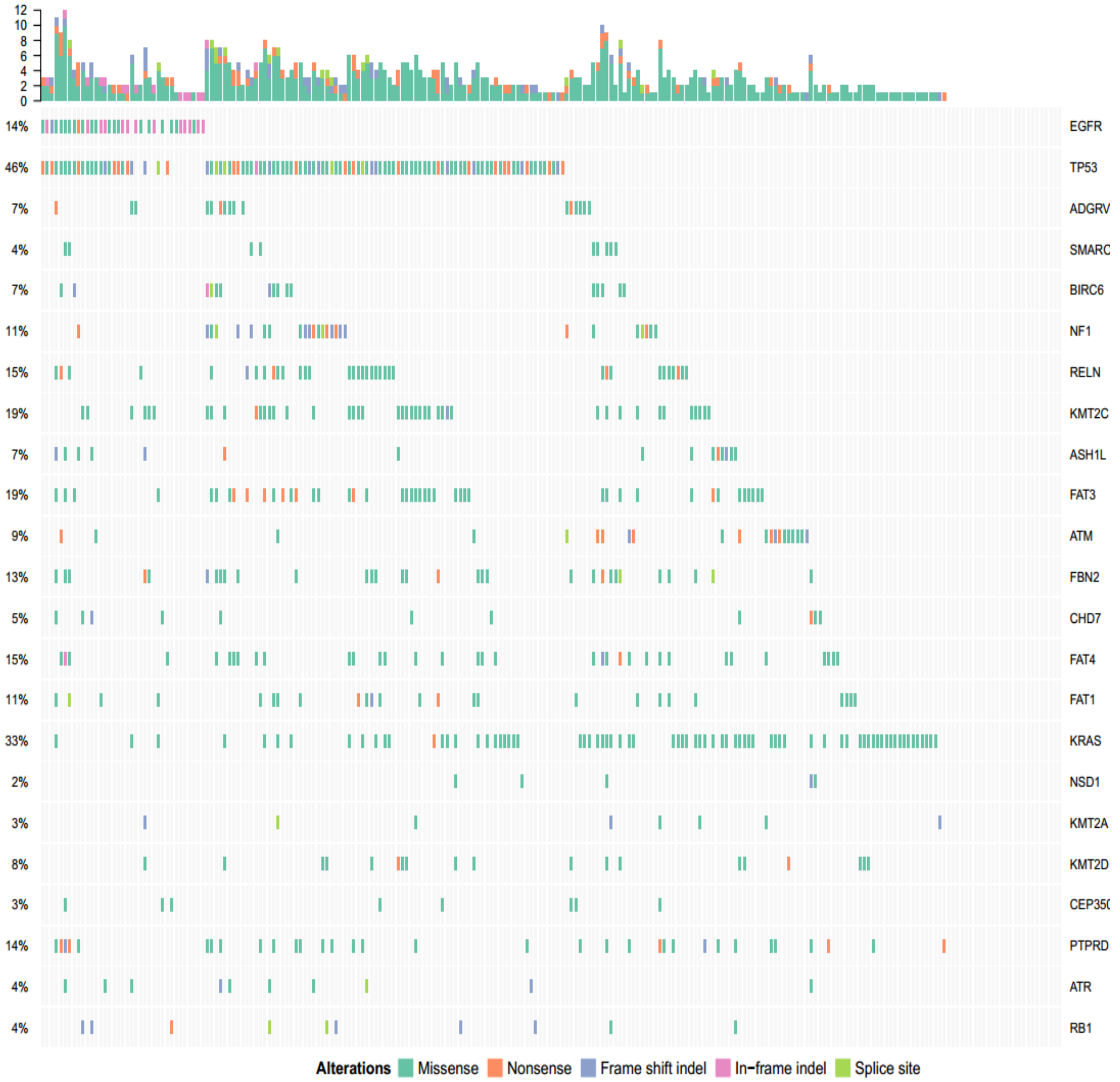

Current study

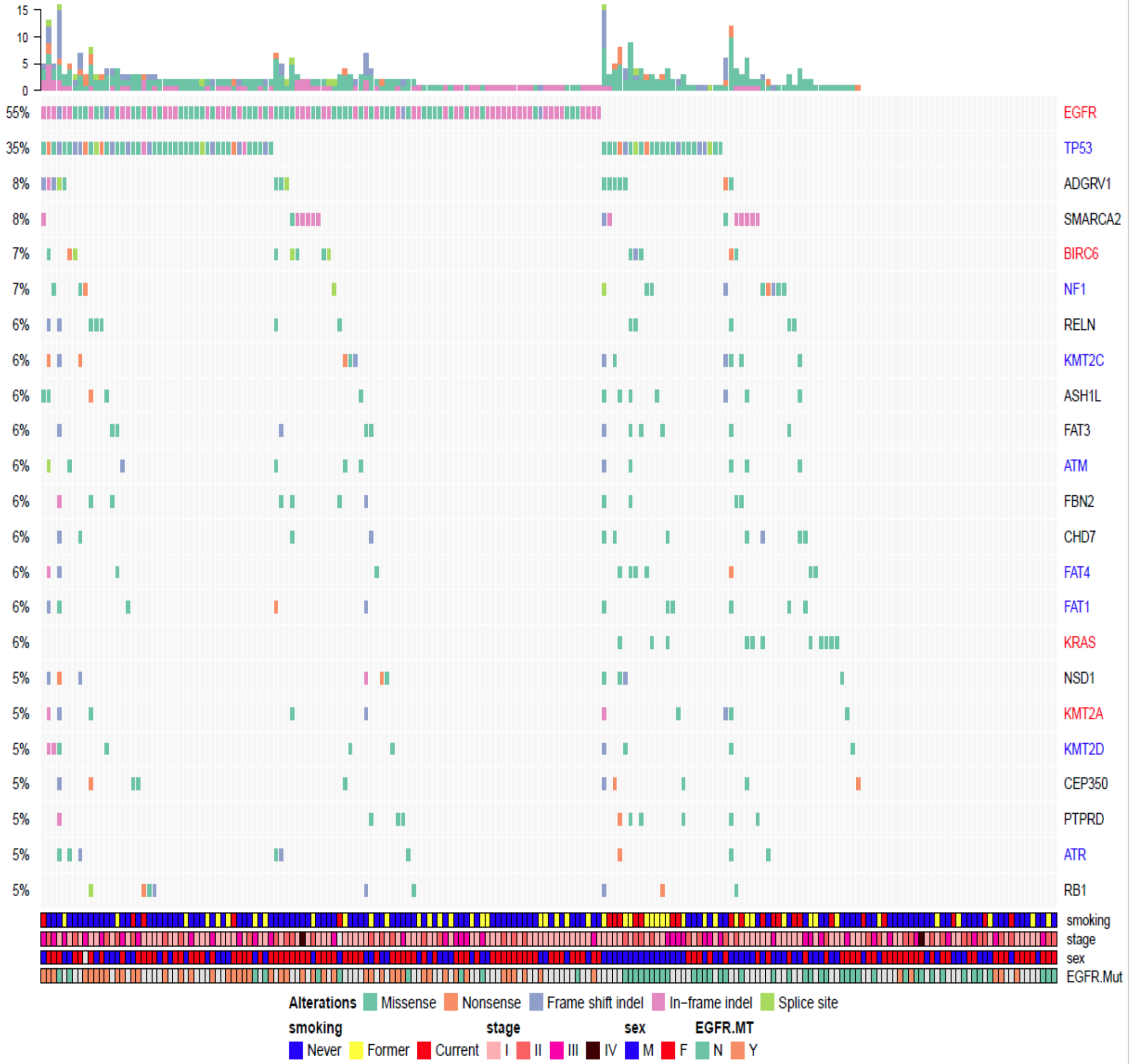

S2BFig. Comparison of *EGFR* hot spot mutation between current study and TCGA data

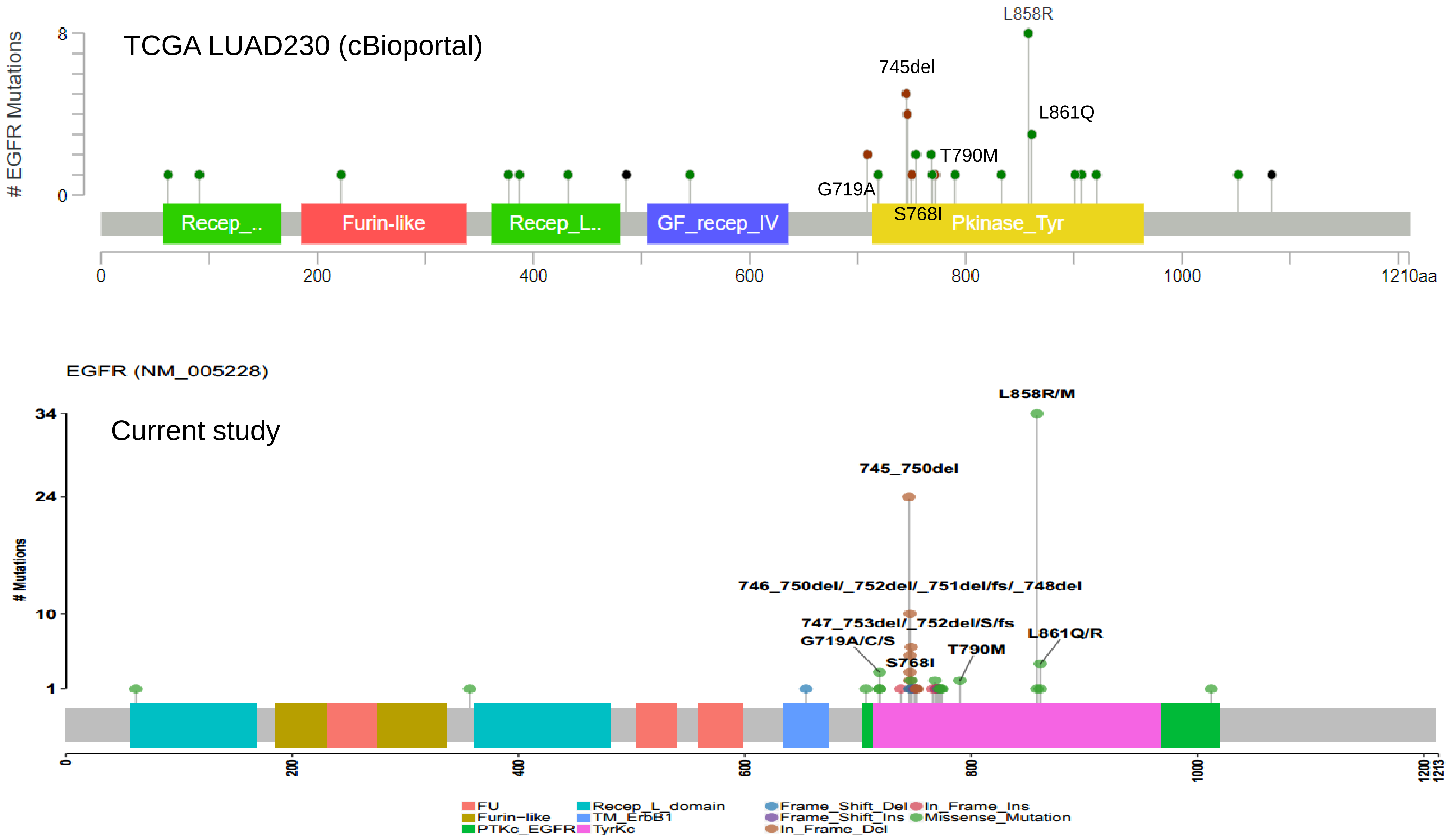

S2CFig. Comparison of *TP53* hot spot mutation between current study and TCGA data

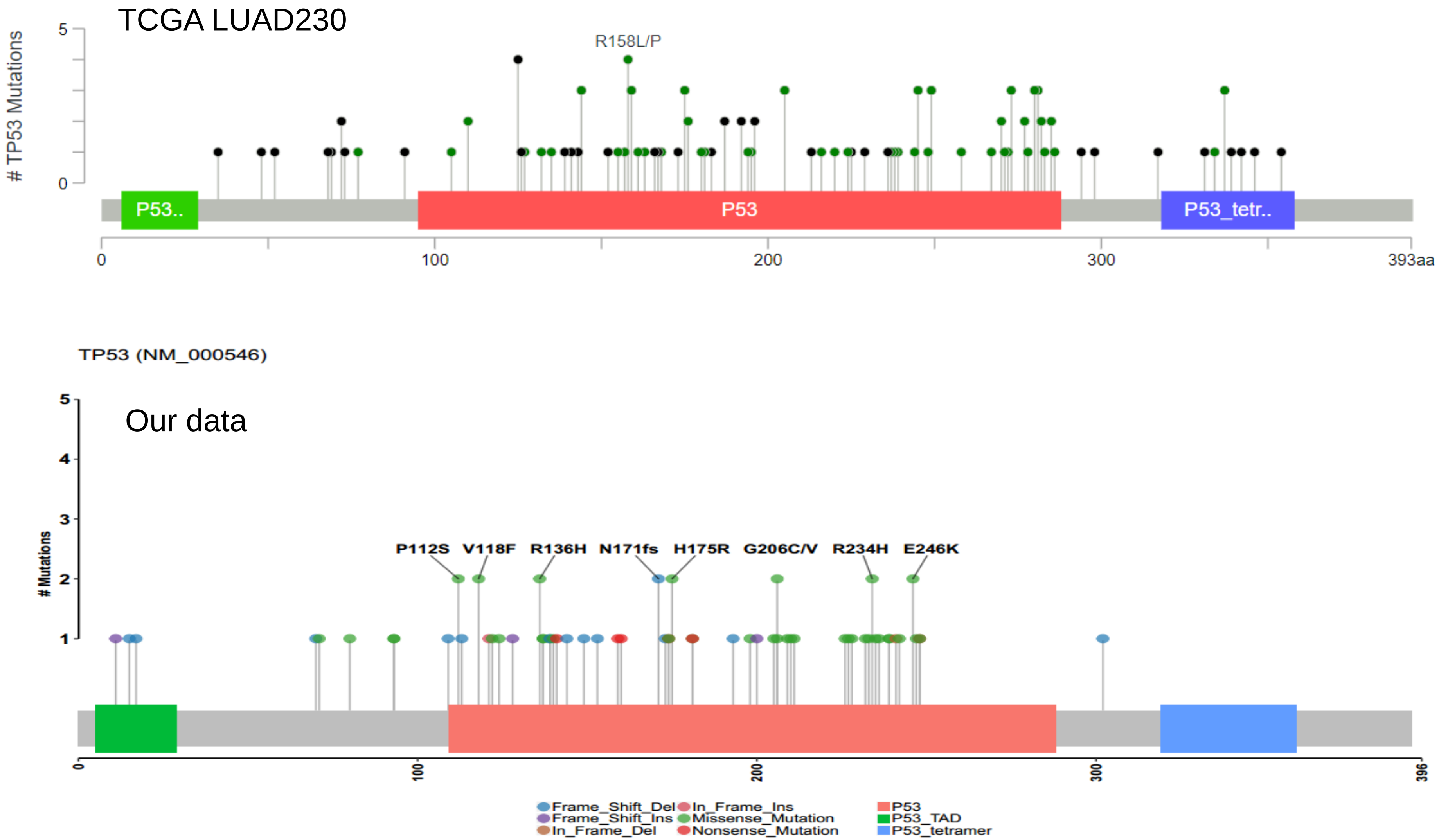

S2DFig. Comparison of *KRAS* hot spot mutation between current study and TCGA data

TCGA LUAD230

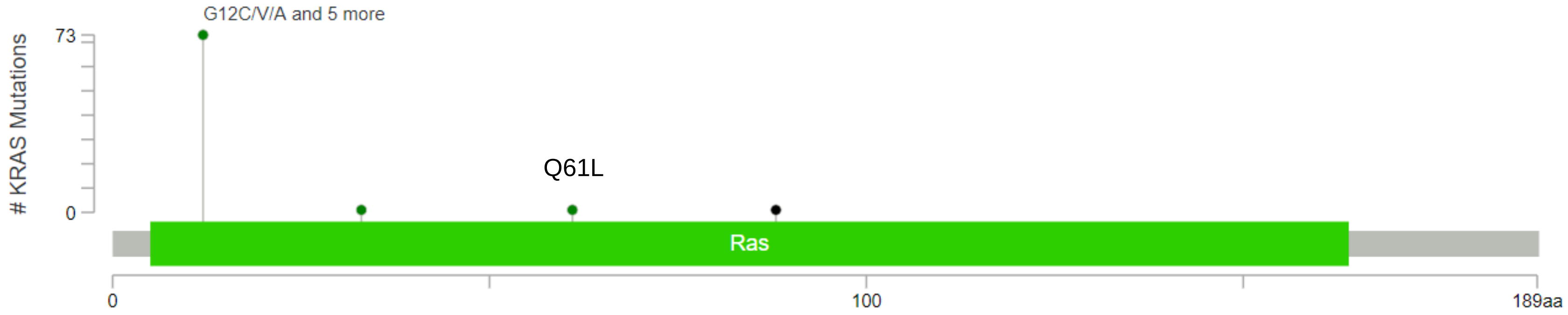

Our data

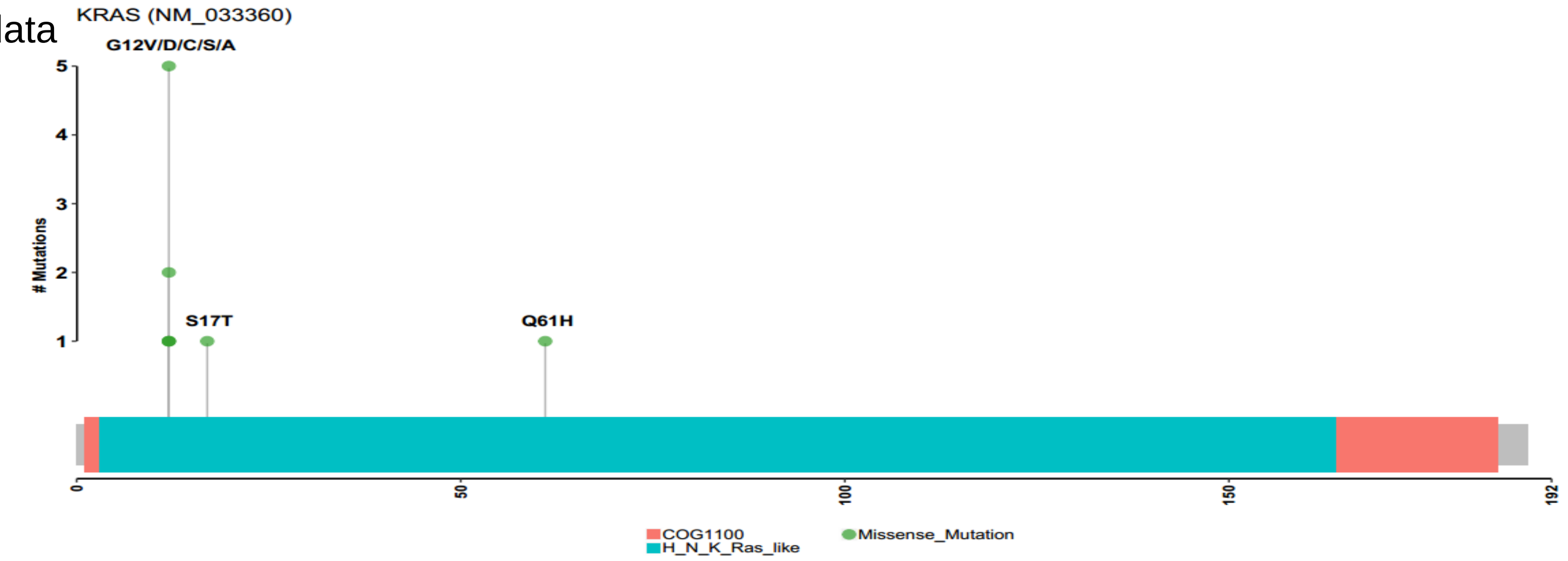

S2EFig. Comparison of *PIK3CA* hot spot mutation between current study and TCGA data

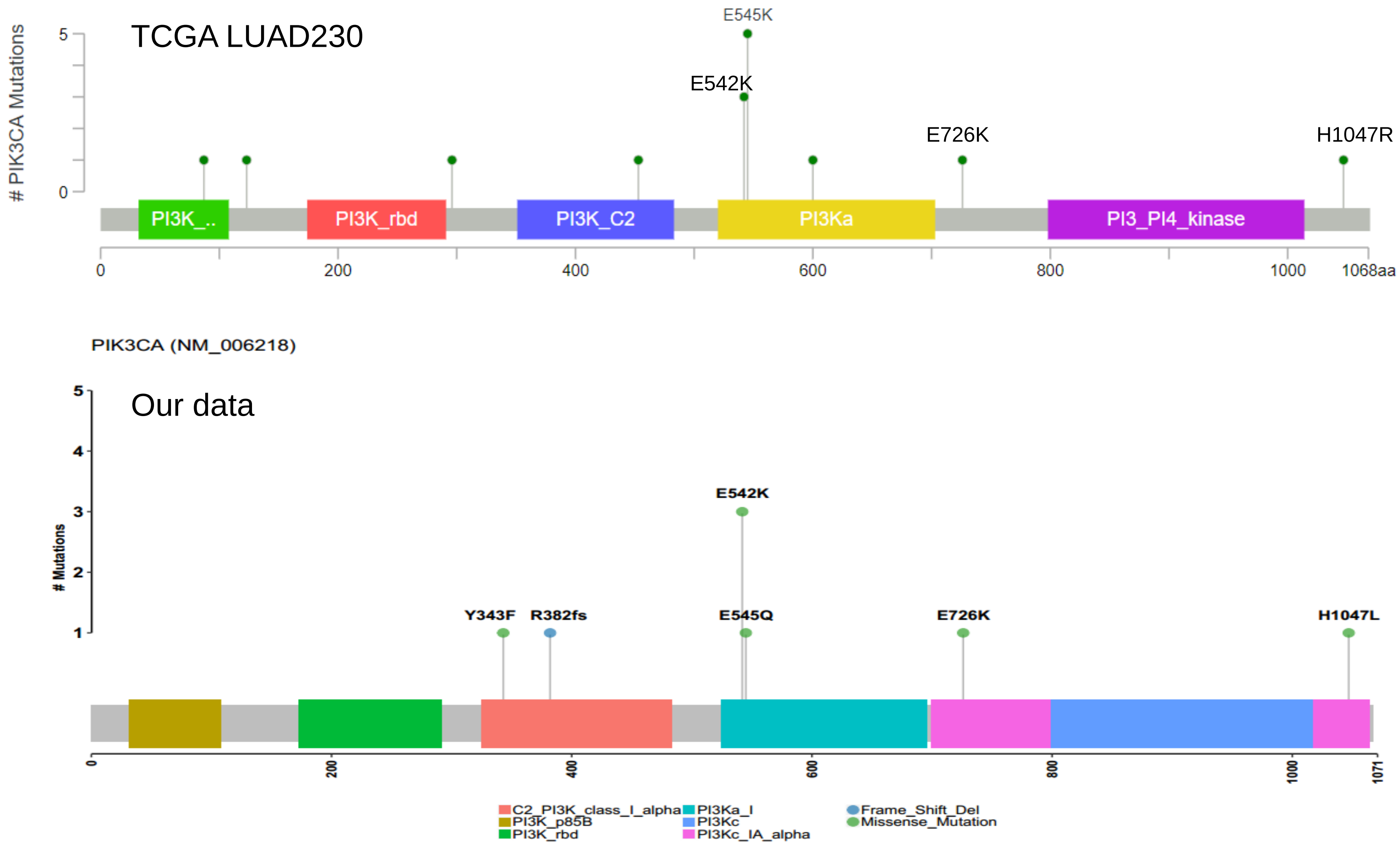

S2FFig. Comparison of CNV between current study and TCGA data

red: oncogene
blue: tumor suppressor gene

TCGA

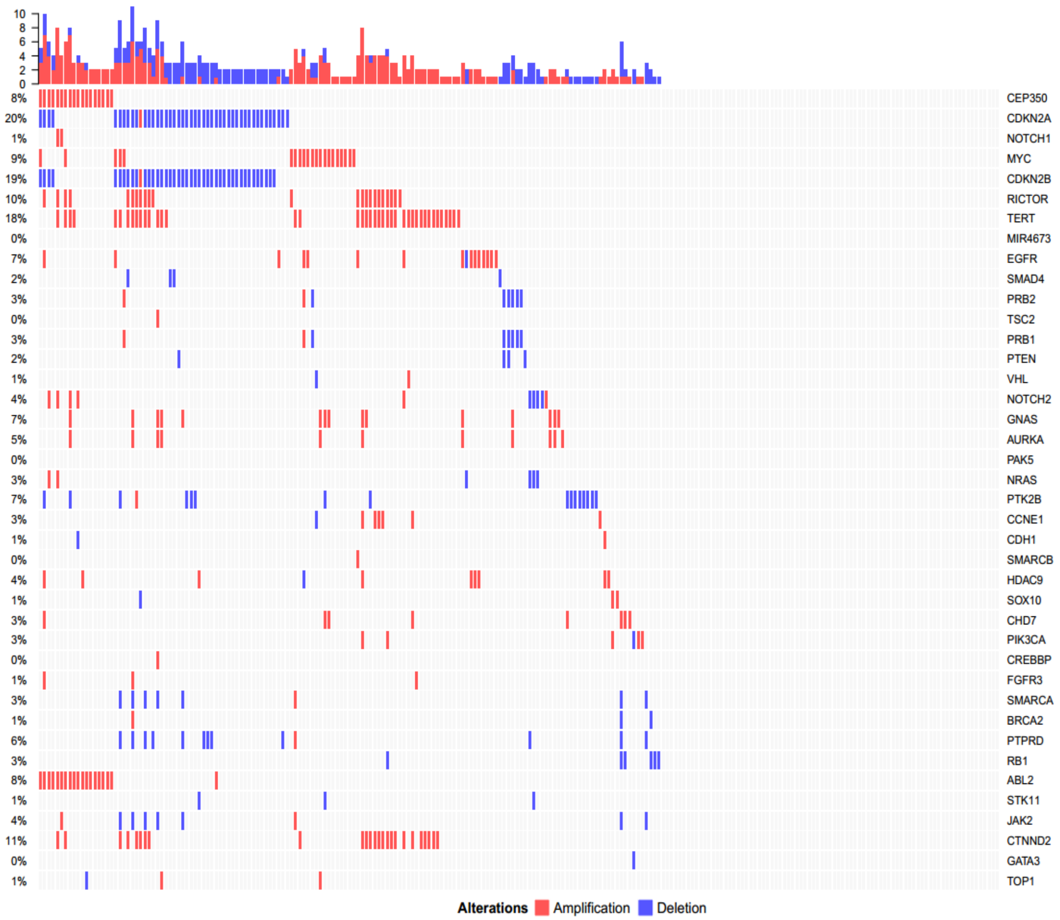

Oncogene : Amplification
TSG : Deletion

Current study

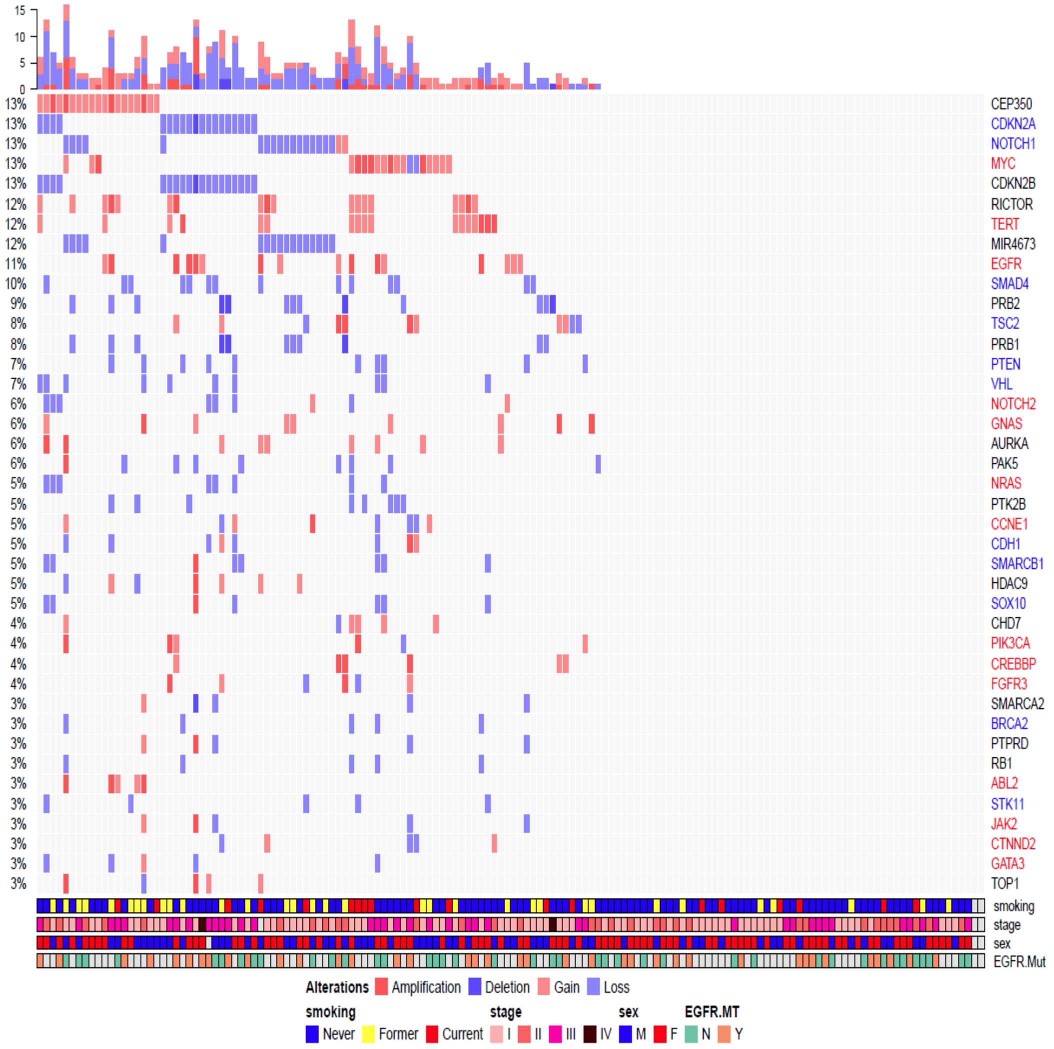

Supplement: S2 Fig — (A) Comparison of SNV between current study and TCGA data. (B) Comparison of EGFR hot spot mutation between current study and TCGA data. (C) Comparison of TP53 hot spot mutation between current study and TCGA data. (D) Comparison of KRAS hot spot mutation between current study and TCGA data. (E) Comparison of PIK3CA hot spot mutation between current study and TCGA data. (F) Comparison of CNV between current study and TCGA data. (PDF) [file pone.0224379.s002.pdf]
