# Supplementary material for: Molecular characterization of lung adenocarcinoma from Korean patients using next generation sequencing
Source: PLoS One. 2019 Nov 25;14(11):e0224379. doi: 10.1371/journal.pone.0224379 (PMC6876835; doi:10.1371/journal.pone.0224379)
Supplement: S5 Fig — Disease-free survival (DFS) by somatic mutations in (A) TP53, (B) EGFR, (C) RTK-RAS pathway, and (D) PI3K pathway. (PDF) [file pone.0224379.s005.pdf]

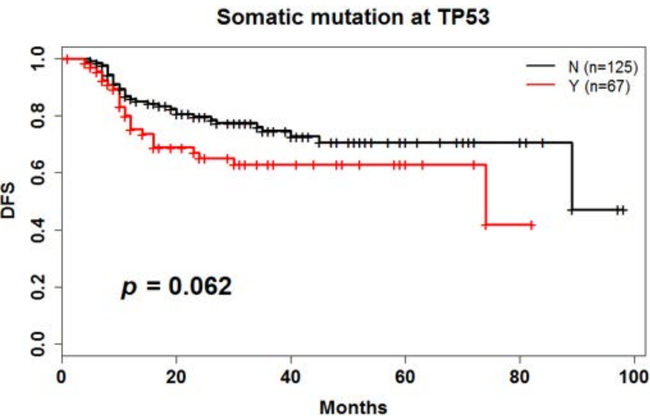

Somatic mutation at TP53
N (n=125)
Y (n=67)
DFS
Months
p = 0.062
0.0
0.2
0.4
0.6
0.8
1.0
0
20
40
60
80
100

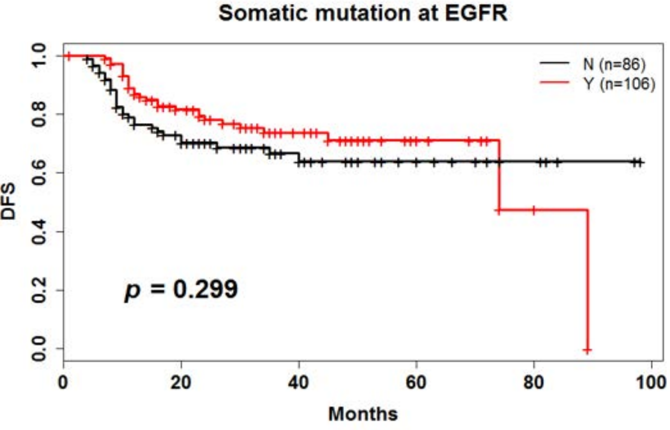

Somatic mutation at EGFR
N (n=86)
Y (n=106)
p = 0.299
DFS
Months
0.0
0.2
0.4
0.6
0.8
1.0
0
20
40
60
80
100

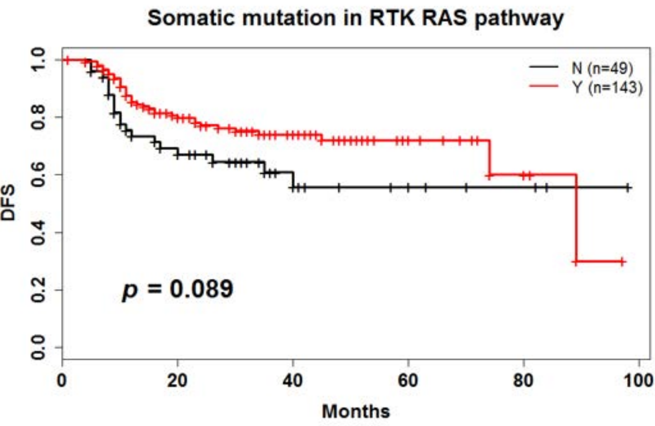

Somatic mutation in RTK RAS pathway
N (n=49)
Y (n=143)
p = 0.089
DFS
1.0
0.8
0.6
0.4
0.2
0.0
0
20
40
60
80
100
Months

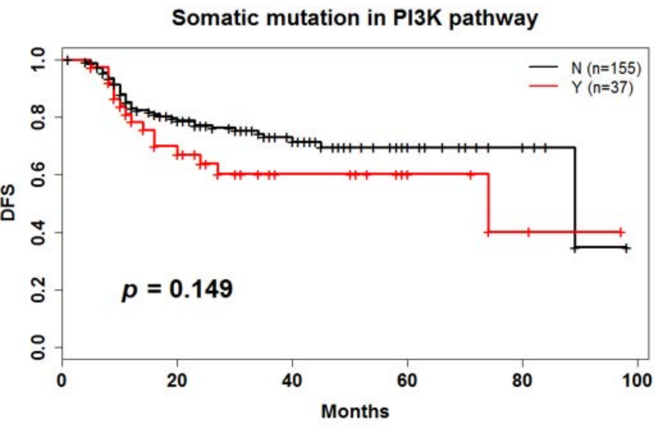

Somatic mutation in PI3K pathway
N (n=155)
Y (n=37)
DFS
1.0
0.8
0.6
0.4
0.2
0.0
p = 0.149
0
20
40
60
80
100
Months
